# NGS Transcriptomes and Enzyme Inhibitors Unravel Complexity of Picrosides Biosynthesis in *Picrorhiza kurroa* Royle ex. Benth

**DOI:** 10.1371/journal.pone.0144546

**Published:** 2015-12-11

**Authors:** Kirti Shitiz, Neha Sharma, Tarun Pal, Hemant Sood, Rajinder S. Chauhan

**Affiliations:** Department of Biotechnology and Bioinformatics, Jaypee University, Waknaghat-73234, Solan, Himachal Pradesh, India; National Taiwan University, TAIWAN

## Abstract

*Picrorhiza kurroa* is an important medicinal herb valued for iridoid glycosides, Picroside-I (P-I) and Picroside-II (P-II), which have several pharmacological activities. Genetic interventions for developing a picroside production platform would require knowledge on biosynthetic pathway and key control points, which does not exist as of today. The current study reports that geranyl pyrophosphate (GPP) moiety is mainly contributed by the non-mevalonate (MEP) route, which is further modified to P-I and P-II through phenylpropanoid and iridoid pathways, in total consisting of 41 and 35 enzymatic steps, respectively. The role of the MEP pathway was ascertained through enzyme inhibitors fosmidomycin and mevinolin along with importance of other integrating pathways using glyphosate, aminooxy acetic acid (AOA) and actinomycin D, which overall resulted in 17%-92% inhibition of P-I accumulation. Retrieval of gene sequences for enzymatic steps from NGS transcriptomes and their expression analysis vis-à-vis picrosides content in different tissues/organs showed elevated transcripts for twenty genes, which were further shortlisted to seven key genes, ISPD, DXPS, ISPE, PMK, 2HFD, EPSPS and SK, on the basis of expression analysis between high versus low picrosides content strains of *P*. *kurroa* so as to eliminate tissue type/ developmental variations in picrosides contents. The higher expression of the majority of the MEP pathway genes (ISPD, DXPS and ISPE), coupled with higher inhibition of DXPR enzyme by fosmidomycin, suggested that the MEP route contributed to the biosynthesis of P-I in *P*. *kurroa*. The outcome of the study is expected to be useful in designing a suitable genetic intervention strategy towards enhanced production of picrosides. Possible key genes contributing to picroside biosynthesis have been identified with potential implications in molecular breeding and metabolic engineering of *P*. *kurroa*.

## Introduction

*Picrorhiza kurroa* Royle ex. Benth is an important medicinal herb valued for its hepatoprotective activity as well as other medicinal properties like anti-malarial, anti-inflammatory, anti-oxidant, anti-bacterial, immune modulator, etc., which are attributed to the presence of iridoid glycosides, Picroside-I (P-I) and Picroside-II (P-II) [1]. The reckless collection of plant material from wild along with unorganized cultivation and low seed viability has led to the endangered status of this important medicinal herb.

Herbal drug formulations have been an integral part of Ayurvedic system of medicine for centuries. With an ever-increasing global demand for herbal medicine, there is not only a demand for large quantity of raw material of medicinal plants, but also of appropriate quality where active compounds are present in desired concentrations [2]. *P*. *kurroa* is used in a number of commercially available drug formulations like livocare, livomap, livplus, katuki, arogya, etc. for different disorders containing combinations of P-I and P-II in different concentrations [1]. P-I and P-II possess different medicinal properties individually as well as in combination and are, therefore, two major constituents of *P*. *kurroa* having therapeutic importance in several herbal drug formulations [3]. P-I is reported to be antimicrobial [4] and used against hepatitis B [5]. P-II possess different pharmacological activities such as antiapoptotic [6], neuroprotective [7], anti-inflammatory [8], anti-oxidant [9] and prevents myocardial ischemia reperfusion injury [10]. The proper concentration and ratio of P-I and P-II are, therefore, important in determining the quality and efficacy of *P*. *kurroa*-based herbal drug formulations.

The biosynthesis and accumulation of P-I and P-II occur differentially in different tissues of *P*. *kurroa*. The biosynthesis of P-I occurs in shoots and P-II in roots or stolons whereas both accumulate in rhizomes [11,12]. Biosynthesis of picrosides occurs through a combined biosynthetic route involving non-mevalonate (MEP), mevalonate (MVA), phenylpropanoid and iridoid pathways. Picrosides are monoterpenoids with iridoid backbone and glycoside moiety. Picrosides are classified as P-I and P-II based on functional group moieties; P-I having cinnmate moiety and P-II having vanillate moiety [13], both derived from phenylpropanoid pathway. Iridoid backbone is derived from geranyl pyrophosphate (GPP) which is synthesized by head to tail condensation of isopentenyl pyrophosphate (IPP) and its allelic isomer dimethylallyl diphosphate (DMAPP) via cytosolic mevalonate (MVA) and/or plastidic non-mevalonate (MEP) pathway [14,15]. Biosynthesis of picrosides involves the synthesis of iridoid moiety from GPP through series of oxidation and cyclization steps followed by condensation of glucose moiety and cinnamate/vanillate from phenylpropanoid pathway [16,17]. The complete biosynthetic pathway of picrosides has been deciphered for all possible intermediates [17], however, the role and contribution of corresponding genes catalysing the enzymatic steps are not known. There are 41 steps (15 from MEP and MVA pathway, 14 from iridoid pathway, 11 from phenylpropanoid pathway, 1 involved in esterification of catalpol) and 35 steps (15 from MEP and MVA pathway, 14 from iridoid pathway, 5 from phenylpropanoid pathway, 1 involved in esterification of catalpol) involved in the biosynthesis of P-I and P-II, respectively. The cinnamate/vanillate moieties are first CoA activated and then transferred to the catalpol for the formation of respective iridoids. Out of 35 steps, 32 till 3-dehydroshikimate are common for both P-I and P-II. After that P-II pathway is diverted for production of vanillic acid (4-hydroxy-3-methoxybenzoic acid) and P-I pathway is diverted for production of cinnamic acid. The final step involved in the esterification of catalpol (iridoid backbone) for the formation of P-I and P-II is common. An alternative route for the formation of vanillic acid by degradation of ferulic acid has also been reported in *Vanilla planifolia* [18] and *Pseudomonas fluorescens* [19]. Various studies have reported the partial biosynthetic pathway for picrosides along with few enzymatic steps. Kawoosa et al [16] reported 15 steps of MEP and MVA pathway with their corresponding enzymes but intermediate steps from GPP till the formation of picrosides were missing. Two genes of phenylpropanoid pathway (4-CH and 3-CH) and involvement of CYPs and glycosyltransferases in picrosides biosynthesis was also reported [20]. Singh et al [13] cloned 8 genes of the MEP and MVA pathways and reported two additional genes (PAL and COMT) of phenylpropanoid pathway. Five remaining genes of MEP and MVA pathway were cloned by Pandit et al [21]. Additionally, cloning of UGT gene of iridoid pathway was done by Bhat et al [22]. Each of these studies has shown that the corresponding enzymatic steps are involved in the biosynthesis of P-I and P-II. However, none has clarifies as to which of the MVA/MEP pathways contribute to the iridoid backbone, GPP and which genes are playing key role in contributing to the biosynthesis of P-I and P-II in *P*. *kurroa*. Multiple pathways contribute in biosynthesis of secondary metabolites, therefore it is necessary to identify the flux of individual pathways for the production of final product. Inhibitor studies can provide significant clues in ascertaining the contribution of individual biosynthetic pathways towards production of terpenoids. Palazon et al [23] showed that non-mevalonate pathway is the main source of universal terpenoid precursor isopentenyl diphosphate (IPP) for biosynthesis of taxanes in *Taxus baccata*. The major involvement of mevalonate pathway for shikonins biosynthesis in *Arnebia euchroma* has also been proved through inhibitor assays [24]. However, any such studies has not been taken up in *P*. *kurroa* for identification of major contributing pathway for picrosides among various integrating pathways.

Present work reports on ascertaining the contribution of MVA and/or MEP route in the biosynthesis of P-I through enzyme inhibitor experiments. Also, genes catalysing the enzymatic steps were mapped to iridoid branch of the picrosides biosynthetic pathway which were not known in *P*. *kurroa*. The availability of NGS transcriptomes of different *P*. *kurroa* tissues enabled the selection of appropriate paralogs for pathway genes. Expression analysis of all genes involved in the complete biosynthetic pathway was carried out in four different tissues of *P*. *kurroa* with varying contents of P-I (0.0% and 2.7%) and P-II (0.0% and 0.4%) to associate key genes involved in picrosides biosynthesis. Further, to ascertain the involvement of genes in picrosides biosynthesis as well as to eliminate the effect of tissue type/developmental stage vis-à-vis picrosides contents, expression analysis of pathway genes was also assesed in high versus low P-I content strains of *P*. *kurroa*.

## Results

### Mapping genes to enzymatic steps of iridoid branch of the pathway

**Fig 1:**
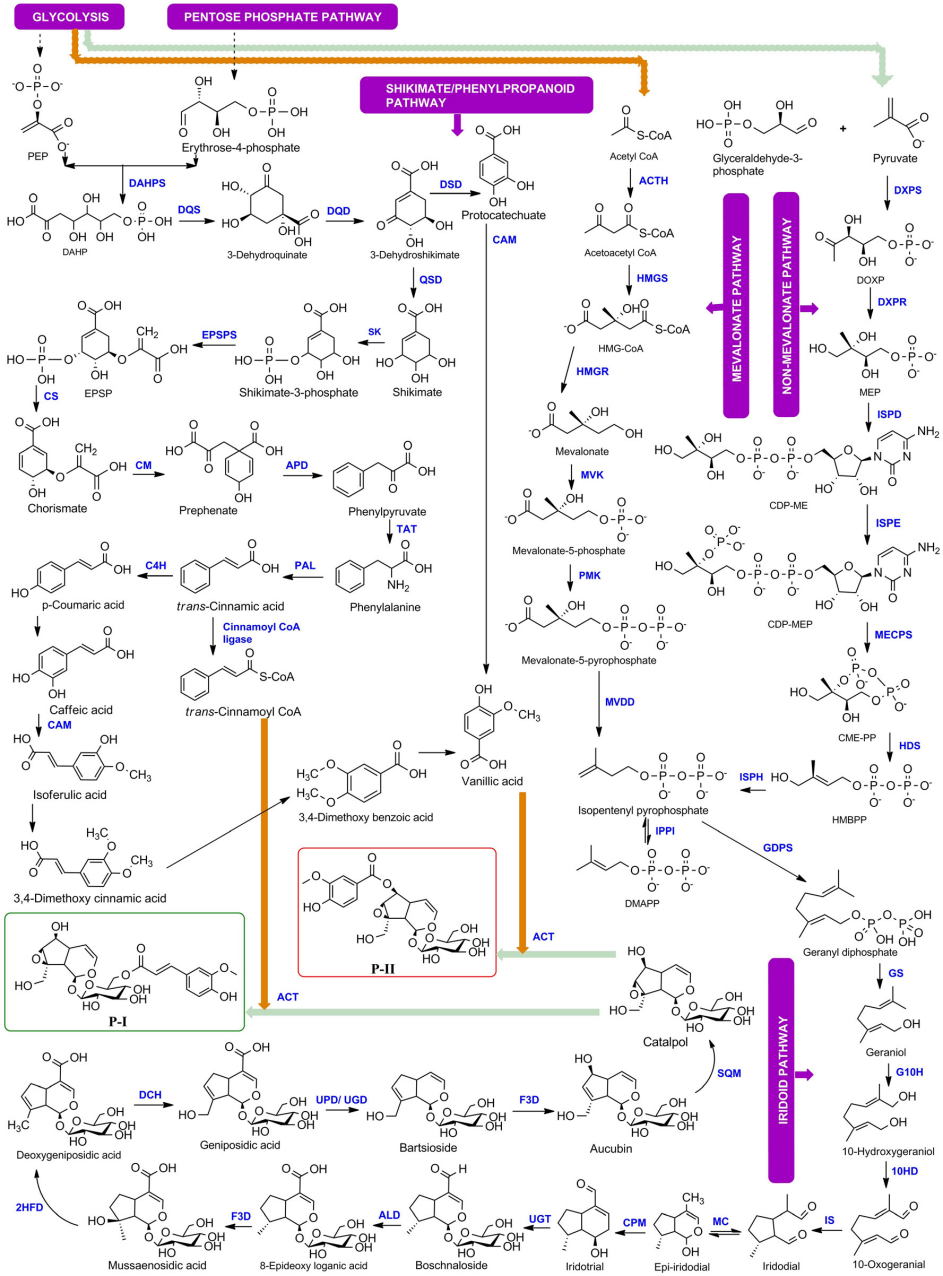
Complete biosynthetic pathway for Picroside-I and Picroside-II of *Picrorhiza kurroa*.

The first seven enzymatic steps GS, G10H, 10HGO (10HD), IS, MC, CPM, UGT of iridoid pathway have recently been identified during this study in medicinal plant species such as *P*. *kurroa*, *Catharanthus roseus* and *Gardenia jasmonides* [22,25,26] while rest of the steps were unknown. The eighth step in the pathway is conversion of boschnaloside to 8-epideoxy loganic acid which involves hydroxylation/oxidation reaction. This enzymatic step is most probably catalysed by aldehyde dehydrogenase (ALD). The ninth step is conversion of 8-epideoxy loganic acid to mussaenosidic acid, which is catalysed by an hydroxylase enzyme. The similar reaction is catalyzed by flavanone 3-dioxygenase/hydoxylase (F3D). The tenth step is conversion of mussaenosidic acid to deoxygeniposidic acid, which is catalyzed by dehydratase enzyme. We searched for all the dehydratases present in *P*. *kurroa* transcriptomes and looked for their involvement in secondary metabolism. 2-hydroxyisoflavanone dehydratase (2HFD) was the only dehydratase involved in secondary metabolites biosynthesis, therefore, it was predicted to be catalysing a similar reaction in *P*. *kurroa*. The eleventh step is conversion of deoxygeniposidic acid to geniposidic acid, which involves hydroxylation reaction. The similar reaction is catalyzed by deacetoxycephalosporin-C hydroxylase. The twelth step is conversion of geniposidic acid to bartsioside, which is catalyzed by a decarboxylase enzyme. Two decarboxylases, uroporphyrinogen decarboxylase (UPD) and udp-glucuronic acid decarboxylase (UGD) were identified from *P*. *kurroa* transcriptomes. Uroporphyrinogen decarboxylase catalyzes the decarboxylation of uroporphyrinogen to coproporphyrinogen. Similar chemical reaction is involved in the conversion of geniposidic acid to bartsioside. The thirteenth step is the conversion of bartsioside to aucubin and this reaction is similar to ninth step, which is being catalyzed by a similar enzyme flavanone 3-dioxygenase/hydroxylase (F3D). Fourteenth step is conversion of aucubin to catalpol which is catalyzed by an epoxidase or monooxygenase enzyme. We searched for all the monooxygenases and epoxidases in *P*. *kurroa* transcriptomes and identified two enzymes squalene monooxygenase (SQM) and squalene epoxidase (SQE) involved in secondary metabolism but squalene epoxidase did not show significant expression w.r.t. P-I or P-II contents. The last step in the formation of P-I and P-II from catalpol involves acyl group transfer reaction and we predicted this reaction to be catalyzed by anthocyanin acyltransferase (ACT). The complete biosynthetic pathway with corresponding enzymatic steps is shown in Fig 1.

### Identification of appropriate paralogs/isoforms

NGS transcriptomes were generated from different *P*. *kurroa* tissues varying in picrosides content mentioned in our previous work [27]. The assembly statistics for transcriptome datasets is given in S1 Table. Mining of transcriptomes for picrosides biosynthetic pathway genes showed the existence of multiple paralogs of these genes. The appropriate paralog for each gene was identified and selected for expression analysis using the methodology described in methods section. The paralogs selected for pathway genes in different shoot transcriptomes of *P*. *kurroa* are given in S2 Table.

### Expression analysis of P-I and P-II biosynthetic pathway genes vis-à-vis picrosides content

**Fig 2:**
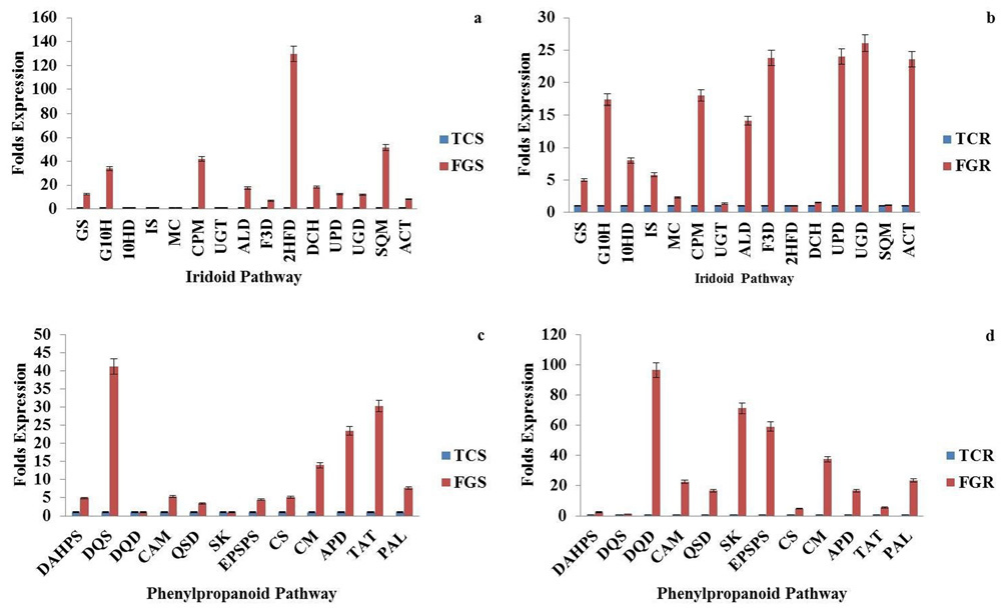
Expression status of iridoid (a, b) and phenylpropanoid (c, d) pathway genes in field grown tissues (FGS: Field grown shoots having 2.7% P-I and FGR: Field grown roots having 0.4% P-II) w.r.t. tissue cultured shoots (TCS having 0.01% P-I) and roots (TCR having 0.0% P-II).

**Fig 3:**
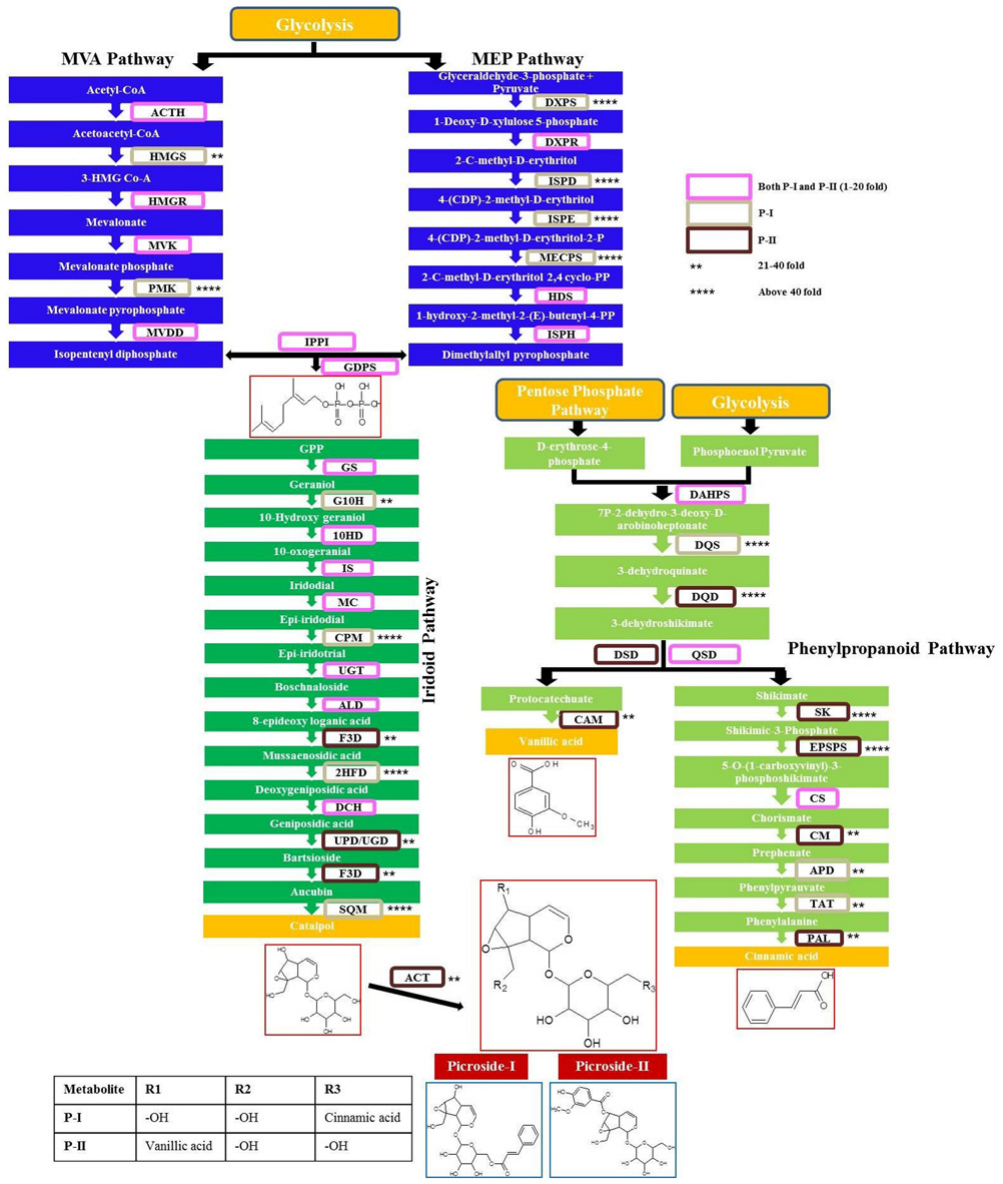
Expression status of all genes depicted as fold increase, for P-I and P-II biosynthetic pathways in *Picrorhiza kurroa*: MVA and MEP pathway genes data from Pandit et al. (2012).

**Fig 4:**
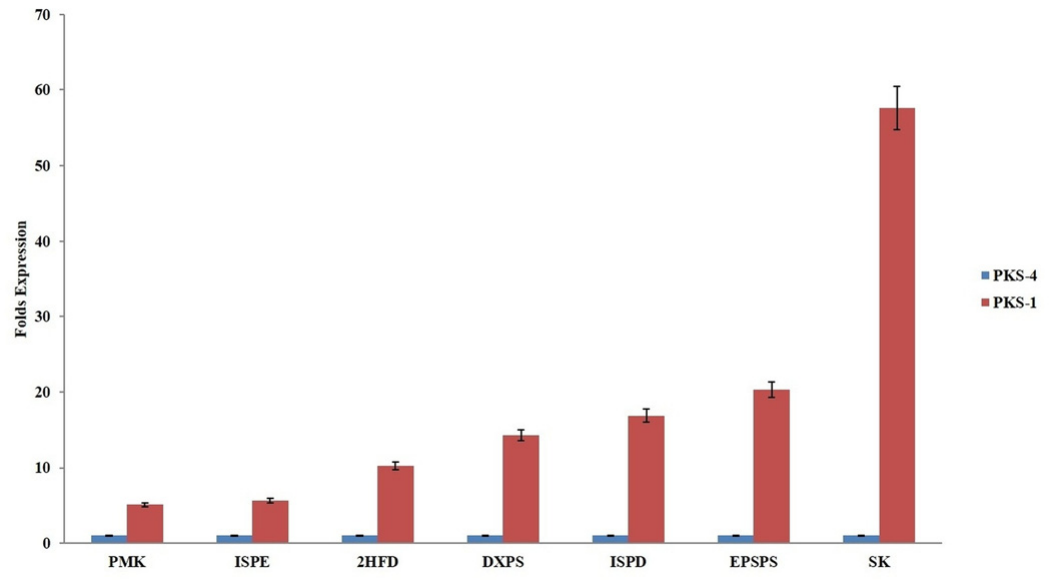
Expression pattern of key genes in shoot tissues of high (PKS-1) versus low (PKS-4) Picroside-I content accessions of *Picrorhiza kurroa*.

The results are in continuation to our previous work which reported the expression status of 15 genes of MEP and MVA pathway in different tissues of *P*. *kurroa* [21]. Expression analysis of remaining genes of the biosynthetic pathway (15 genes from iridoid pathway and 12 from phenylpropanoid pathway) was done in four tissues varying for P-I and P-II contents. Majority of the genes showed higher expression in relation to picrosides content. Four genes of iridoid pathway (GS, 2HFD, DCH and SQM) and two genes of phenylpropanoid pathway (DQS and TAT) showed elevated levels of transcripts (~12–130 folds) in FGS having 2.7% P-I content w.r.t. TCS having negligible (0.01%) P-I. Two genes of iridoid pathway (F3D and ACT) and six genes of phenylpropanoid pathway (DQD, QSD, CAM, SK, EPSPS and PAL) showed ~16–96 folds expression in FGR having 0.4% P-II content wr.t. TCR having no P-II content. Four genes of iridoid pathway (G10H, CPM, ALD and UPD/UGD) and two genes of phenylpropanoid pathway (CM and APD) showed ~12–42 folds higher expression in FGS as well as FGR w.r.t. their tissue cultured counterparts. The difference in fold expression in field grown tissues in comparison to tissue culture grown for iridoid and phenypropanoid pathways is given in Fig 2a, 2b, 2c and 2d. The overall expression status of pathway genes w.r.t. P-I and P-II is given in Fig 3. The genes which showed above 10 folds transcript abundance in initial screening w.r.t. either P-I or P-II or both (including MEP/MVA pathway genes from our previous study) were checked for their expression status on shoot tissues of high versus low P-I content strains of *P*. *kurroa*. A high P-I content strain, PKS-1 (2.7%) and a low content strain, PKS-4 (0.3%) were used in comparative expression analysis. This resulted in the identification of seven genes, ISPD, DXPS, ISPE, PMK, 2HFD, EPSPS, SK from the complete biosynthetic pathway with ~5–57 folds higher transcript abundance in high content strain compared to low content strain of *P*. *kurroa* (Fig 4). The expression of three genes of MEP pathway, DXPS, ISPE, ISPD, one gene of MVA pathway, PMK and one gene of iridoid pathway, 2HFD decreased from 57–160 folds to ~5–17 folds when compared for their relative transcript abundance between strains versus different tissue samples. The expression level of three genes of MEP pathway ISPD, DXPS and ISPE decreased from 160, 57 and 99 folds to 16.8, 14.2 and 5.6 folds, respectively in high content strain. The expression of *PMK* gene of MVA pathway decreased from 107.6 fold to 5.1 fold. 2HFD of iridoid pathway decreased from 130 to 10.2 fold (Table 1). The relative expression status of pathway genes between high versus low content strains thus provided a realistic association with the biosynthesis of picrosides.

**Table 1:** Folds difference in expression status of key genes between A (FGS vs TCS) and B (PKS-1 vs PKS-4).

Gene	A	B	Fold Difference
PMK	107.66	5.10	-21.1
ISPE	99	5.63	-17.6
2HFD	130	10.25	-12.7
DXPS	57	14.25	-4.0
ISPD	160	16.86	-9.5
EPSPS	4.5	20.28	+4.5
SK	1.03	57.59	+55.9

### Effect of enzyme inhibitors on biosynthesis of Picroside-I

**Fig 5:**
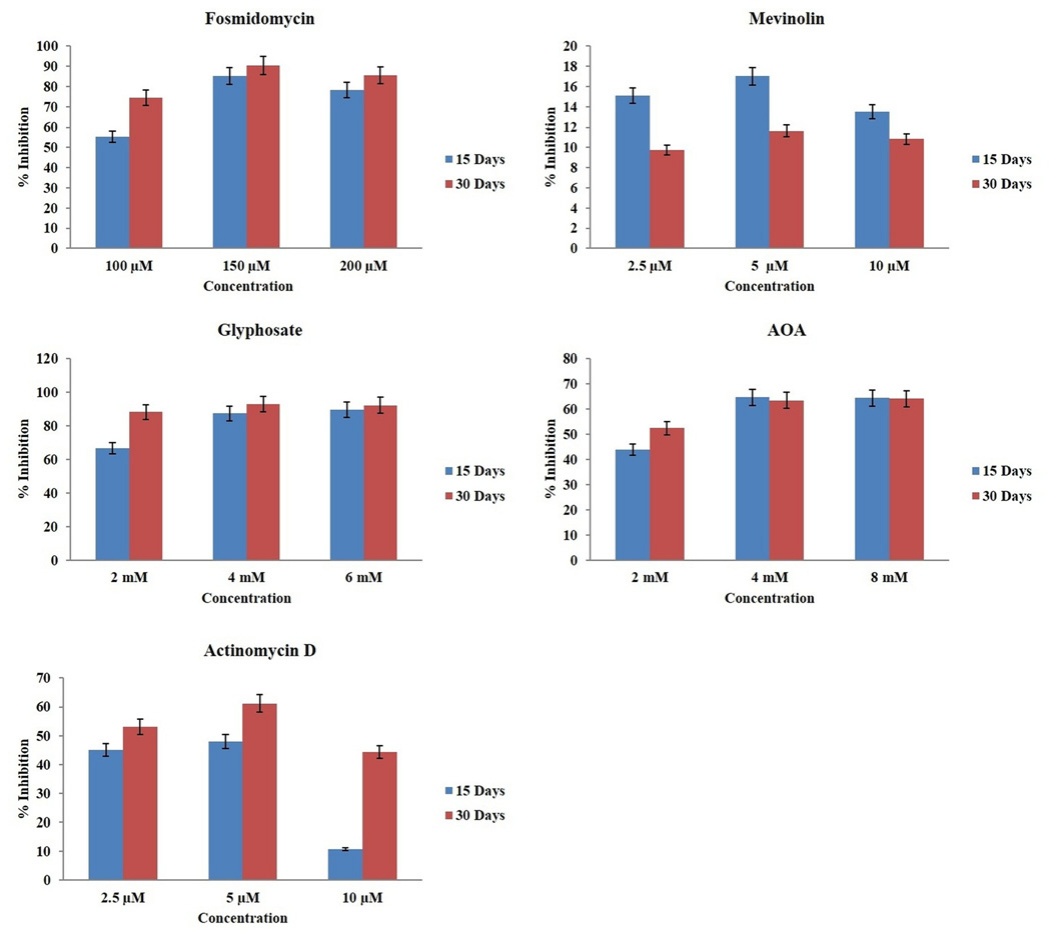
Inhibition profiles of enzyme inhibitors on Picroside-I biosynthesis in *Picrorhiza kurroa*.

The inhibition profile of five inhibitors was assessed at 15^th^ and 30^th^ day of culturing *P*. *kurroa* shoots by quantifying P-I content (Fig 5) because cultured shoots are reported to accumulate only P-I [11]. Out of five inhibitors, mevinolin, fosmidomycin, glyphosate and AOA are competitive inhibitors which are highly specific for their target enzymes while actinomycin D is a transcriptional inhibitor. The best inhibitory concentrations were 5 μM for mevinolin and actinomycin D, 150 μM for fosmidomycin, 4 mM for glyphosate and AOA. Mevinolin was found to be least effective in inhibiting P-I biosynthesis as it showed maximum inhibition of only 17% and 11.6% at 15^th^ and 30^th^ day, respectively. Other inhibitors showed upto 92% inhibition in P-I content in comparison to control. All inhibitors showed higher inhibition at 30^th^ day in comparison to 15^th^ day, except mevinolin which unlike others showed higher inhibition at 15^th^ day. At their best inhibitory concentrations, fosmidomycin, glyphosate and AOA showed a drastic reduction of 85.3%, 87.3% and 64.6% respectively in P-I accumulation at 15^th^ day and reduction of 90.6%, 92.9% and 63.4%, respectively at 30^th^ day. Fosmidomycin, glyphosate and AOA were thus found to be potent inhibitors of P-I biosynthesis. Actinomycin D moderately inhibited P-I biosynthesis in *P*. *kurroa* as it showed 47.9% and 61.2% inhibition at 15^th^ and 30^th^ day, respectively.

### Gene expression in response to inhibitors

**Fig 6:**
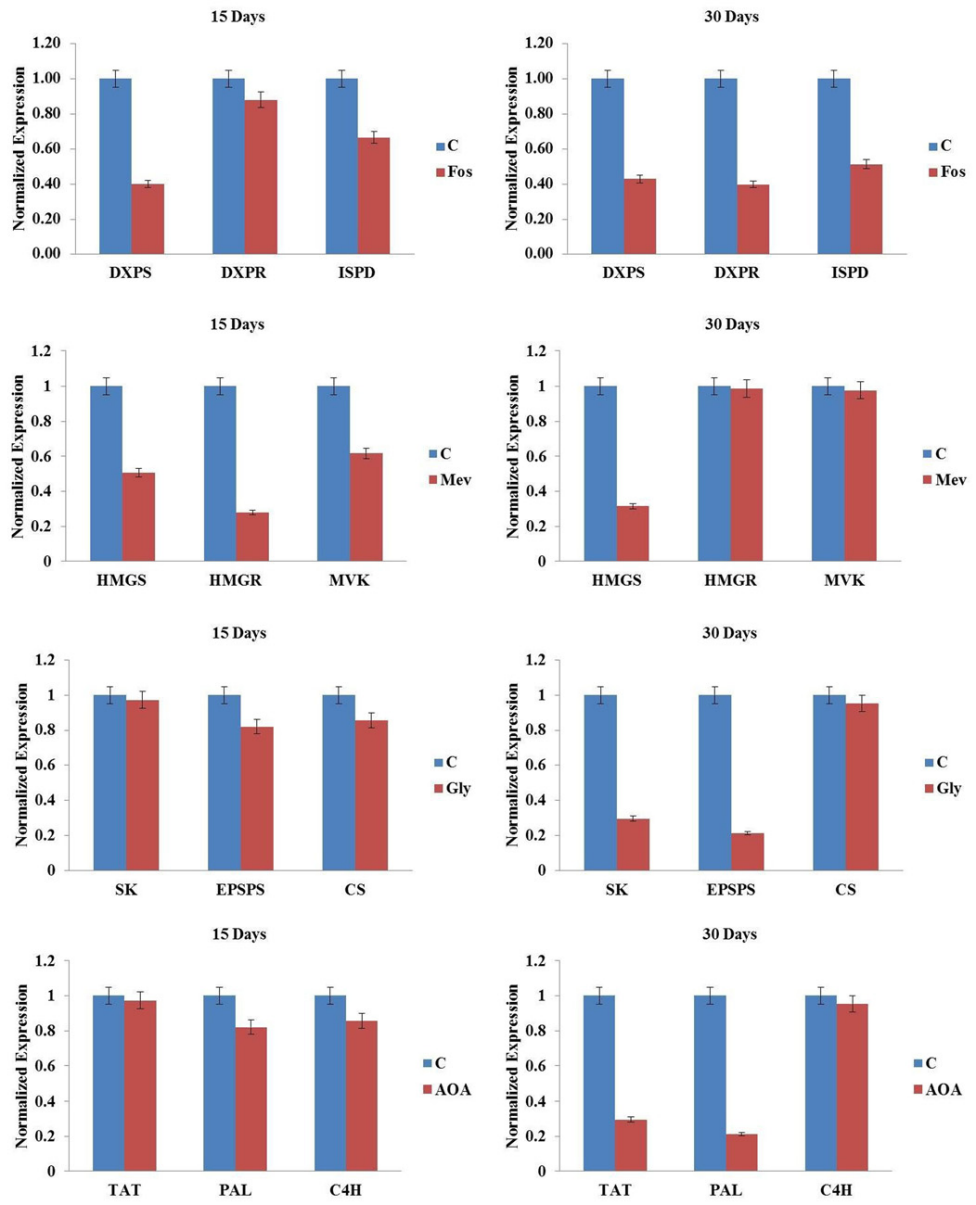
Effect of inhibitors, fosmidomycin, mevinolin, glyphosate and AOA on transcript levels of target as well as upstream and downstream genes in Picroside-I biosynthesis in *Picrorhiza kurroa*.

**Fig 7:**
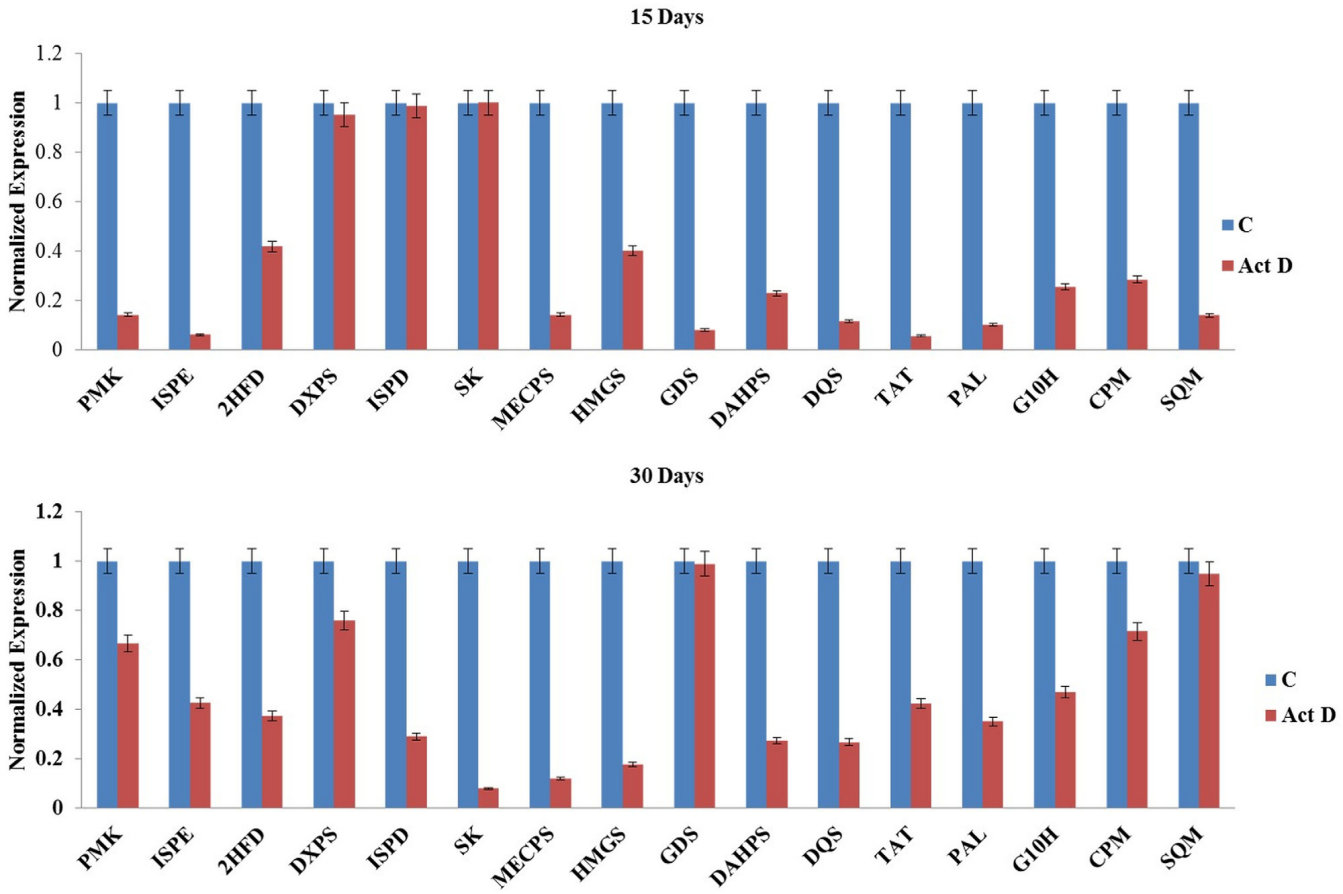
Effect of actinomycin D on expression of significant genes of Picroside-I biosynthetic pathway in *Picrorhiza kurroa*.

To see the effect of inbitor treatments on target gene transcripts, expression analysis was carried out on inhibitor treated shoot tissues in comparison to untreated control for related genes (DXPR, HMGR, EPSPS and PAL) alongwith upstream and downstream genes (1 each). The expression analysis was carried out at the best inhibitory concentrations of 150 μM (fosmidomycin), 5 μM (mevinolin), 4 mM (glyphosate and AOA), and 5 μM (actinomycin D) at 15^th^ and 30^th^ day. The inhibitors fosmidomycin, mevinolin, glyphosate and AOA did not show significant reduction in the transcript levels of their target steps or upstream/downstream genes. Fosmidomycin showed 1.25–2.5 folds reduction in transcript levels in comparison to control at both 15^th^ and 30^th^ day. Mevinolin, glyphosate and AOA also showed similar gene expression pattern with mevinolin (1–3.5 folds), glyphosate (1–4.7 folds) and AOA (1–4 folds) reduction in transcript levels as compared to control (Fig 6). Actinomycin D showed somewhat higher reduction in transcripts from 1 to 17.5 folds (Fig 7).

## Discussion

Through this study MEP pathway has been identified as the major contributor of geranyl pyrophosphate (GPP), the iridoid backbone for picrosides biosynthesis in *P*. *kurroa*. After GPP biosynthesis, iridoid and phenylpropanoid pathways play equally important role in picrosides biosynthesis by providing catalpol and cinnamate/vanillate moieties, respectively. The importance of iridoid and phenylpropanoid pathways has been justified by precursor feeding studies in *P*. *kurroa*, where exogenous feeding of catalpol and cinnamic acid showed that both pathways act in conjunction for increased production of P-I in *P*. *kurroa* [Kumar et al. 2015, unpublished].

Current study is an initial endeavor towards deciphering the complete biosynthetic pathway including corresponding enzymatic steps for picrosides biosynthesis in *P*. *kurroa*. The identification of enzymatic steps of iridoid branch of the pathway, not known earlier has been done, however functional characterization is required to support their proposed functions in *P*. *kurroa*. The availability of NGS transcriptomes of different *P*. *kurroa* tissues enabled the selection of appropriate paralogs for pathway genes as multiple paralogs were present for these genes. The higher expression of gene transcripts for corresponding enzymes vis-à-vis P-I and P-II contents and their involvement in secondary metabolism in other plant species suggested their possible role in catalysing the required enzymatic reactions in picrosides biosynthesis. Aldehyde dehydrogenase is reported to be involved in the biosynthesis of ferulic acid and sinapic acid in *Arabidopsis thaliana* [28]. Flavanone 3-dioxygenase/hydoxylase (F3D) is involved in flavanoids biosynthesis in many plant species like *Ginkgo biloba*, *Camellia sinensis* [29,30]. 2-hydroxyisoflavanone dehydratase is involved in isoflavone biosynthesis in *Lotus japonica* [31]. Deacetoxycephalosporin-C hydroxylase is involved in cephalosporin biosynthesis [32]. Uroporphyrinogen decarboxylase is associated with the activity of enzymes involved in tetrapyrrole biosynthesis and pathogen defense response in *Nicotiana tabacum* [33,34]. Squalene epoxidase/monooxygenase is involved in ginsenoside biosynthesis in *Panax ginseng* [35,36] and triterpenoids in *Uncaria tomentosa* [37]. Anthocyanin acyltransferases catalyze regiospecific acyl transfer from acyl-CoA to the sugar moiety of anthocyanins [38] and are involved in anthocyanin biosynthesis in plant species [39]. The identified enzymes belong to cytochrome P450 family which possess broad substrate specificity i.e. same enzyme can bind to different substrates but enzyme kinetics vary considerably from one substrate to another. Therefore, these enzymes inspite of preferably using other substrates can also catalyse the similar reactions for picrosides biosynthesis using pathway intermediates as their substrates.

The expression analysis revealed that most of the genes of picrosides biosynthetic pathway had relatively higher expression in field grown tissues of *P*. *kurroa* containing higher amounts of P-I or P-II contents compared to tissue culture grown plants with negligible or no picrosides content at all. Genes, GS, G10H, CPM, ALD, F3D, 2HFD, DCH, UPD/UGD, SQM, ACT from iridoid pathway and DQS, DQD, QSD, SK, EPSPS, CM, APD, TAT, PAL from shikimic acid/phenylpropanoid pathway showed significantly higher folds expression vis-à-vis picrosides content. Various studies have reported expression of multiple genes of biosynthetic pathways to be positively correlated with the terpenoid biosynthesis; shikonins in *Arnebia euchroma* [24], artemisinin in *Artemisia annua* [40], MIAs in *Catharanthus roseus* [41], flavonoids biosynthesis in *Fagopyrum* species [42], lignin biosynthesis in *Arabidopsis thaliana* [43]. Geraniol synthase (GS) is an important enzyme which initiates monoterpenoid branch of the pathway in *Catharanthus roseus* [44] and G10H is reported to be a rate-limiting enzyme for biosynthesis of TIAs in *Catharanthus roseus* [45] alongwith its importance in iridoid monoterpenoid swertiamarin biosynthesis in *Swertia mussotii* [46]. EPSPS, SK and PAL are also reported to be important regulatory enzymes of shikimate/phenylpropanoid pathway [47–49]. In initial analysis, multiple genes showed higher expression for P-I or P-II or both. But the differential expression in field grown tissues in comparison to tissue cultured plants might be due to tissue type or environmental variations. Therefore, to ascertain whether the elevated levels of transcripts of pathway genes are only affecting the biosynthesis of picrosides uniquely, the expression status of genes was further studied between shoots of *P*. *kurroa* genotypes that were varying for Picroside-I. High and low Picroside-I content genotypes of *P*. *kurroa* (one each) grown for 3 years in the controlled environment in greenhouse were used for comparative expression analysis so as to reflect genetic differences contributing to the increase or decrease of gene transcripts rather than tissue type or developmental stage. It was observed that most of the genes did not show significant difference in expression between high versus low content strains, thereby suggesting that all the genes which showed higher expression initially might be contributing to the biosynthesis of secondary metabolites other than picrosides. This additional analysis shortlisted seven genes, PMK, ISPE, 2HFD, DXPS, ISPD, EPSPS and SK with major contribution in picrosides biosynthesis. Among these seven genes, four of MEP/MVA pathway ISPD, DXPS, ISPE and PMK are reported to be positively correlated with picrosides content in *P*. *kurroa* [21] and DXPS is a well known regulatory enzyme of MEP pathway [50,51]. EPSPS is a key regulatory gene of shikimate pathway [46] which is associated with herbicide tolerance [52,53] and biosynthesis of secondary metabolites [54,55]. SK is an important regulatory gene in secondary metabolism as it has been suggested that plant SKs act as regulatory points for the shikimate pathway [48]. It has been reported that 2-hydroxyisoflavanone dehydratase plays a key role in the regulation of isoflavone biosynthesis as its overexpression resulted in accumulation of daidzein and genistein in *Lotus japonicas* [31]. Majority of these key genes belonged to MEP pathway. Also higher expression of two genes of shikimic acid/phenylpropanoid pathway and one gene of iridoid pathway, among seven genes, highlights the importance of these modules of the biosynthetic pathway. This suggests that each module of the pathway is important in contributing to the structures of parental compounds, P-I and P-II in *P*. *kurroa*.

Five inhibitors targeting important enzymatic steps of the picrosides biosynthetic pathway were selected to assess their effect on picrosides accumulation. The inhibitor concentrations were chosen based on previous reports [56–59], [23]. Mevinolin and fosmidomycin are highly specific inhibitors of MVA (HMGR) and MEP (DXPR) pathways, respectively [60,61]. These two inhibitors were selected to rule out whether MVA and/or MEP pathway contributes in picrosides biosynthesis. Fosmidomycin produced drastic inhibition of upto 90.6% in P-I accumulation whereas mevinolin resulted in slight (17%) inhibition. This suggested that the MEP pathway plays major role in the production of GPP, the precursor for iridoid backbone biosynthesis. Picrosides are monoterpenoids and our results are in accordance with the previous findings that monoterpenoids have non-mevalonate (plastidial) origin and monoterpenoid synthases are localized to plastids [62]. It has also been reported by Eisenreich et al. [63] that MEP pathway is a predominant contributor for monoterpenoid biosynthesis, however, crosstalk occurs between two pathways [64]. Our previous reports also suggested the predominant role of MEP pathway in picrosides biosynthesis as majority of the MEP pathway genes were highly expressed in relation to picrosides content [21]. Sood and Chauhan [11] have also highlighted the importance of plastids (chloroplasts) by showing that the biosynthesis of Picroside-I occurs only in *in vitro* cultured leaf and stem segments but not in undifferentiated callus cultures.

The other inhibitors, glyphosate and AOA were selected targeting the shikimate/phenylpropanoid pathway enzymes whereas actinomycin D is a common transcriptional inhibitor. Glyphosate, a broad spectrum herbicide which competitively inhibits EPSPS [65] and AOA acts as an inhibitor of PAL [58] which is an important regulatory enzyme in phenylpropanoid pathway. Shikimic acid/phenylpropanoid pathway has a major contribution in picrosides biosynthesis, thereby, providing cinnamate and vanillate moieties for P-I and P-II, respectively. The decrease in P-I biosynthesis by inhibiting shikimic acid/phenylpropanoid pathway enzymes confirms major contribution of this component of the pathway in picrosides biosynthesis in *P*. *kurroa*. Glyphosate is reported to inhibit secondary metabolites content in soyabean and buckwheat [57,66]. AOA treatment resulted in decreased accumulation of phytoalexins in banana [59] and 2-hydroxy-4-methoxybenzaldehyde in *Hemidesmus indicus* roots [67].

The inhibition pattern was similar for four inhibitors fosmidomycin, glyphosate, AOA and actinomycin D where higher inhibition was observed at 30^th^ day than 15^th^ day for all concentrations. Mevinolin showed opposite inhibition pattern as higher inhibition was observed at 15^th^ day than 30^th^ day. This might be due to the fact that mevinolin is not a strong inhibitor in our case. Therefore, most of it is getting used up till 15^th^ day, hence not showing significant inhibition at 30^th^ day.

The inhibitor treatment thus resulted in decrease in picrosides biosynthesis. We further looked at whether the expression of biosynthetic pathway genes, which are targets of corresponding inhibitors, is getting affected or not. Competitive inhibitors did not show significant decrease in expression of transcripts because these might be affecting the genes only at enzymatic level but are not producing any effect at the transcriptional level. It has been reported in *Arabidopsis* that inhibitor treatment did not show significant effect on the expression of genes involved in sterol, chlorophyll and carotenoid metabolism, thereby indicating that posttranscriptional processes might be playing an important role in regulating the flux through isoprenoid metabolic pathways [68].

## Conclusion

The knowledge of complete pathway and corresponding genes would be helpful in understanding molecular basis of picrosides biosynthesis as well as planning genetic improvement strategies for enhancing picrosides content in *P*. *kurroa*. Inhibitor experiments revealed that MEP pathway is a major contributor of GPP for picrosides in *P*. *kurroa* and picrosides biosynthesis is regulated at various control points in different modules of the biosynthetic route. Therefore, common regulatory elements for multiple genes need to be identified so as to ease genetic interventions for enhancing picrosides content. This is the first report wherein possible key genes have been identified in picrosides biosynthetic pathway which after further functional validation can have potential implications in molecular breeding and metabolic engineering.

## Materials and Methods

### Plant material

*P*. *kurroa* plants were procured from the nursery of Himalayan Forest Research Institute, Jagatsukh, Manali, H.P., India (1,900 m altitude, 20°35.6′–32°6.1′N and 78°57.8′–77°33.7′E) and maintained in the greenhouse of Jaypee University. These plants were cultured and maintained in an optimized Murashige and Skoog (MS) medium [69] supplemented with 3 mg/L indole-3-butyric acid and 1 mg/L kinetin in a plant tissue culture chamber maintained at 25±2°C with 16 h photoperiod provided by cool white fluorescent light (3,000 lux) (Sood and Chauhan 2010). Four *P*. *kurroa* tissues with varying picrosides content growing in field conditions and tissue culture were taken for the study (Table 2, [21]). These included field grown shoots having 2.7% P-I (FGS), tissue cultured shoots having negligible P-I content (TCS), field grown roots having 0.4% P-II content (FGR) and tissue cultured roots with no P-II content (TCR). Two strains of *P*. *kurroa* with high and low content viz. PKS-1 (P-I 2.7%) and PKS-4 (P-I 0.3%) maintained in controlled environment in green house were also taken.

**Table 2:** Description of *Picrorhiza kurroa* tissues used for qRT-PCR analysis.

Sample Name	Description	Picroside-I %	Picroside-II %
FGS	Field grown shoots	2.7	-
TCS	Tissue cultured shoots grown at 25°C	0.01	-
FGR	Field grown roots	-	0.4
TCR	Tissue cultured roots grown at 25°C	-	0.0
PKS-1	High content strain	2.7	-
PKS-4	Low content strain	0.3	-

### Identification of unknown enzymatic steps

The genes catalysing the enzymatic steps of iridoid pathway were identified following the methodology described below. Initially the enzyme classes were predicted on the basis of chemical reactions/group transfer involved in the conversion of one metabolite to the next. All possible enzymes catalysing similar reactions were searched in the KEGG database. The enzymes were then matched in *P*. *kurroa* transcriptomes using BLAST and shortlisted on the basis of significant similarity. Next, the enzymes were shortlisted based on their functionality i.e. involved in secondary metabolites biosynthesis. The enzymes were then selected on the basis of abundance (FPKM) of enzyme transcripts in transcriptomes and finally supported through qRT-PCR in different tissues of *P*. *kurroa* with varying contents of P-I and P-II. The nucleotide sequences for phenylpropanoid and iridoid pathway genes were extracted from whole genome transcriptomes of *P*. *kurroa* and primers were designed using online tool Primer3.

### RNA isolation and cDNA synthesis

Total RNA was isolated from *P*. *kurroa* tissues by using RaFlex^™^ total RNA isolation kit (GeNei^™^) by following manufacturer’s instructions. The quality of RNA was checked by 1% (w/v) ethidium bromide-stained agarose gel. RNA was quantified in a NanoDrop spectrophotometer (Thermo Scientific) by measuring absorbance at 260nm and 280nm and 1μg RNA was used for cDNA synthesis. cDNA was synthesized using Verso cDNA synthesis kit (Thermo Scientific) according to manufacturer’s protocol. Concentration of each cDNA sample was adjusted to 100ng/μl for qRT-PCR.

### Identification of appropriate paralogs/isoforms for pathway genes in transcriptomes

NGS transcriptomes for *P*. *kurroa* were generated and analysed using the methodology represented in S1 Fig Multiple paralogs of the genes were present in *P*. *kurroa* transcriptomes, therefore appropriate paralogs/isoforms were identified for each gene. For selection of suitable paralogs of genes, initially multiple sequence alignment of the selected transcripts using ClustalW was carried out to see the homology within paralogs for each gene. Further, shortlisting of transcripts was done through BLAST analysis by looking for highest similarity with functionally characterized sequences (retrieved from the NCBI databases which were functionally characterized in the same plant or in different plant species). Thereafter, the transcripts were selected on the basis of transcript abundance (FPKM values), in different transcriptomes generated from *P*. *kurroa* tissues varying in picrosides contents. The selected paralogs were further validated through qRT-PCR analysis under differential conditions of picrosides biosynthesis.

### Expression analysis using quantitative real time PCR (qRT-PCR)

*P*. *kurroa* tissues with varying P-I and P-II contents were selected for correlating the expression status of pathway genes with picrosides content. Equal cDNA quantities (100ng) of each sample were taken. Gene specific primers were checked initially on cDNA for amplification of single product using standard PCR. Quantitative real time PCR was performed using using gene specific primers (Table 3) in triplicate on a CFX96 system (Bio-Rad Laboratories; Hercules CA) with the iScript one step RT PCR kit (Bio-rad). The PCR protocol was as follows: denaturation for 3 min at 95°C, followed by 39 cycles each of denaturation for 10 s at 95°C, annealing for 30 s at 50–65°C, and elongation for 20 s at 72°C. In final step melt curve analysis was done at 65–95°C with 0.5°C increment at each 0.05 s to verify amplification of a single product. Two housekeeping genes, 26s rRNA and GAPDH were used as internal controls for normalization. Genes with significantly high expression w.r.t. P-I and P-II content were checked for their expression status in high versus low picrosides content strains (one each) of *P*. *kurroa* to validate/ascertain the genes playing significant role in picrosides biosynthesis. Finally, the expression status of target genes in response to treatment with their specific inhibitors was also checked on *in vitro* cultured shoots of *P*. *kurroa* w.r.t. their untreated controls.

**Table 3:** Gene specific primers used for qRT-PCR.

Genes	FP	RP	Annealing Temp (°C)	Fragment size (bp)
26S	CACAATGATAGGAAGAGCCGAC	CAAGGGAACGGGCTTGGCAGAATC	58	500
GAPDH	TTGCCATCAATGACCCCTTCA	CGCCCCACTTGATTTTGGA	56	215
ACTH	AGTGTTACTAGAGAGGAGCAGGACA	CCTAGACCTTCATCCTTATCAACAA	50	110
HMGS	GATGGTGCAAGAAAAGGCAACTAGA	GGATATTCACTGGCAAGATTGGGCT	54	110
HMGR	CGTTCATCTACCTTCTAGGGTTCTT	GACATAACAACTTCTTCATCGTCCT	60	100
MVK	ATTAACTCTGAGTATGACGGGTCTG	GAGAGCCCATTTATTTAGCAACTC	50	110
PMK	TGGATGTTGTCGCATCAGCACCTGG	GTAATAGGCAGTCCACTCGCTTCAA	58	100
MVDD	GTAACTCTGGATCCTGACCACCT	TAATACCCCCTCTTTTTCATCCTC	54	100
IPPI	TCTCCTATTCACTGTAAGGGATGTT	ACCACTTAAACAAGAAGTTGTCCAC	54	110
GDS	GATATATGTTCTGAGGGAATGGATG	ATACACCTAGCGAAATTCCTCAACT	55	110
DXPS	ACATTTAAGTTCAAGTCTGGGAGTG	ATGTGCACTCTCTTCTCTTTTAGGA	55.9	110
DXPR	GGAGGAACTATGACTGGTGTTCTT	CAGGTCATAGTGTACGATTTCCTCT	54.9	110
ISPD	GAGAAAAGTGTATCTGTGCTTCTTAG	AATAACCTGCGGTGTATGCATTTCC	56	150
ISPE	TTCATCTAGATAAGAAGGTGCCAAC	CCTCTACCAGTACAATAAGCAGCTC	55	110
MECPS	ATCTATAGCGGCAAACCTACAC	ACTTTAGAGAGGGATGGAGGG	57.1	110
HDS	ATGCCATTTAAGGAACTTGCAACAG	GGAGCACCACCAACATATCCAAATT	58	110
ISPH	CATACTGGGTTGACAGTGATGTAAG	TAAGGACATCTTCAACAGCCTTATC	57.2	110
GS	TGGGTAGATTAGAAGCCAGA	CTGGTGATTTCTACCAGCTC	52	139
G10H	TATCGAGCTTTTCAGTGGAT	GATGTGAGTCCTGTCGATTT	52	136
10HD	GGTAGTGTTTATTGGTGCAG	GATCAACTGATCAAGGTCAA	54	172
IS	AATAAGGCCTTGGTTTATCC	TTAGCCTTAGGATCAACTGC	49	116
MC	AAGGCTGCTCGTACCGATAA	AATGGGGCAGTAGAGTGGTG	58.5	132
CPM	ACCTGAGAGATGGCTAGATG	AGTTCACAGGTTTGTGTTCA	53	188
UGT	AATGGTTGGACTCACAAGAG	ACAAGAAGGTCTGGTTGCTA	55	126
ALD	ACTTTGCTGTGGGATTAAGT	TTCCACAAACTAGAGCAATG	57	174
F3D	AAGAATATGGCTTCTTCCAG	ATCCCTCCAATTATGAACAG	55	197
2HFD	ATTTACTCGACAGGATGTGG	AGCACAAATCAACACCTTCT	52	134
DCH	CATTTCACATCTCCACTGAG	ATGGACAAAGCTTCTTTAGC	53	194
UPD	CTGACGGCGTTATTATTTTC	TCTTCAGAACGGATAGGAGA	51	115
UGD	CATGTTGGACCTTTCAATCT	TGGGCCTGAACTCTATCTTA	51	109
SQM	GTTGATATACCCGGTCAGAA	TTTCTCAACAGCAGACATGA	51	126
ACT	TATGTCCAAGAATCCAGTGT	AAGAACTCGTCTCGTTCAA	55	111
DAHPS	ACACCATTAAAGCTCCTTGT	TAACAGTCTGAGATCCACCA	59	171
DQS	TGTTCAAAGTGTTGGTGTCT	GTGACATGTGTGGATATGCT	54	192
DQD	AAAAACAACCAGCTTCAAAT	TCTTTGAAACACAATCGAAA	55	193
CAM	GAAGATGCTCCTTCTTATCC	AACACTCGACCAGAATCAC	53	187
QSD	CATAGTTTGGTTCCTCCTCT	ACTGTCTTTCTAGCCCAGAT	52	186
SK	TATGGTGAGAGCTTCTTCAG	AGTTCCGACAGCTGTAATC	56	195
EPSPS	CACAGAACTCAGAAAGTTGG	AGTAATCAGGGAAGGTCTTG	48.5	203
CS	CTCGCTACCAGTCATAAAAG	GAGTGTGTGGTGATTCTGTT	49	185
CM	GTCTACACACCTGCCATTAG	GTACAAATCAGCAACTAGGC	52	198
APD	GCGTATTGGCTATAGAACTG	CGACCTCTGAGAGTAGTTGA	50	192
TAT	CTACATTGAGATCGACTTCC	ACTATCTCCTGAGGACGTTT	53	206
PAL	GCAAGATAGATACGCTCTAA	GTTCCTTGAGACGTCAAT	49	136
C4H	GCAACATTGATGTTCTCAAC	TCCAGCTCTTCAAGGACTAT	53	169

### Inhibitor treatment and picrosides quantification

Five inhibitors Mevinolin (HMGR), Fosmidomycin (DXPR), Glyphosate (EPSPS), Actinomycin D (transcriptional inhibitor) and Aminooxyacetic acid-AOA (PAL) inhibiting different enzymatic steps (mentioned above in braces) of MEP, MVA and phenylpropanoid pathways were purchased from Sigma-Aldrich, USA. To prepare stock solutions mevinolin and actinomycin D were dissolved in DMSO, rest of the inhibitors were dissolved in autoclaved distilled water and filter sterilized using 0.22 μm sterile filters (Millipore). Shoot apices of length 1 cm were cut from six weeks old cultures of *P*. *kurroa* shoots raised *in vitro* at 25°C and cultured in test tubes containing 10 ml agar gelled MS media containing different concentrations of inhibitors. Inhibitors were added separately at three different concentrations as follows: Mevinolin and actinomycin D (2.5 μM, 5 μM, 10 μM), fosmidomycin (100 μM, 150 μM, 200 μM), glyphosate (2 mM, 4 mM, 6mM) and AOA (2 mM, 4 mM, 8 mM). Two controls were also used, one having only agar gelled MS media and hormones another having equivalent concentration of DMSO as used for dissolving mevinolin and actinomycin D. After inoculation cultures were incubated at 15±2°C. Shoots were harvested at 15^th^ and 30^th^ day for quantification of picrosides. Estimation of picrosides was done using RP-HPLC following the method described by Sood and Chauhan (2010). The experiment was done in triplicates.

## Supporting Information

S1 Fig Workflow of *de novo* whole transcriptome analysis for *P*. *kurroa* tissues. (TIF)

S1 Table Assembly statistics for *P*. *kurroa* tissue datasets. PKS-25 (Tissue culture grown shoots at 25°C), PKSR (Field grown root), PKSTS (Field grown stolon), PKR-25 (Tissue culture grown roots at 25°C), PKSS (Field grown shoot), PKS-15 (Tissue culture grown shoots at 15°C).
(DOCX)

S2 Table The paralogs selected for pathway genes in different transcriptomes generated from *P*. *kurroa* shoot tissues. (DOCX)

## Abbreviations

ACTH: Acetoacetyl-CoA thiolase
HMGS/R: Hydroxymethyl glutaryl CoA synthase/reductase
MVK: Mevalonate kinase
PMK: Phosphomevalonate kinase
MVDD: Mevalonate diphosphate decarboxylase
IPPI: Isopentenyl pyrophosphate isomerase
GDPS: Geraniol diphosphate synthase
DXPS/R: 1-deoxy-D-xylulose 5-phosphate synthase/reductase
ISPD: 2-C-methylerythritol 4-phosphate cytidyl transferase
ISPE: 4-(cytidine-50-diphospho)-2-Cmethylerythritol kinase
MECPS: 2-C-methylerythritol-2,4-cyclophosphate synthase
HDS: 1-hydroxy-2-methyl-2-(E)-butenyl 4-diphosphate synthase
ISPH: 1-hydroxy-2-methyl-2-(E)-butenyl 4-diphosphate reductase
GS: Geraniol synthase
G10H: Geraniol 10-hydroxylase
10HD: 10-hydroxygeraniol dehydrogenase
IS: Iridoid Synthase
MC: Monoterpene Cyclase
CPM: Cytochrome P-450 monooxygenase
UGT: Uridine diphosphate glycosyltransferase
ALD: Aldehyde dehydrogenase
F3D: Flavanone 3-dioxygenase
2HFD: 2-hydroxyisoflavanone dehydratase
DCH: Deacetoxycephalosporin-C hydroxylase
UPD: Uroporphyrinogen decarboxylase
UGD: UDP-glucuronic acid decarboxylase
SQM: Squalene monooxygenase
ACT: Anthocyanin acyltransferase
DAHPS: 3-Deoxy-D-arabinoheptulosonate 7-phosphate synthase
DQS: Dehydroquinate synthase
DQD: Dehydroquinate dehydratase
DSD: Dehydroshikimate dehydratase
CAM: Caffeic acid-3-o-methyltransferase
QSD: Quinate/shikimate dehydrogenase
SK: Shikimate kinase
EPSPS: 5-enolpyruvylshikimic acid-3-phosphate synthase
CS: Chorismate synthase
CM: Chorosmate mutase
APD: Arogenate/prephenate dehydratase
TAT: Tyrosine aminotransferase
PAL: Phenylalanine ammonia lyase
C4H: Cinnamate 4 hydroxylase
FPKM: Fragments Per Kilobase Of Exon Per Million Fragments Mapped
NGS: Next generation sequencing
