# Supplementary figures and images for: NGS Transcriptomes and Enzyme Inhibitors Unravel Complexity of Picrosides Biosynthesis in Picrorhiza kurroa Royle ex. Benth
Source: PLoS One. 2015 Dec 11;10(12):e0144546. doi: 10.1371/journal.pone.0144546 (PMC4687646; doi:10.1371/journal.pone.0144546)

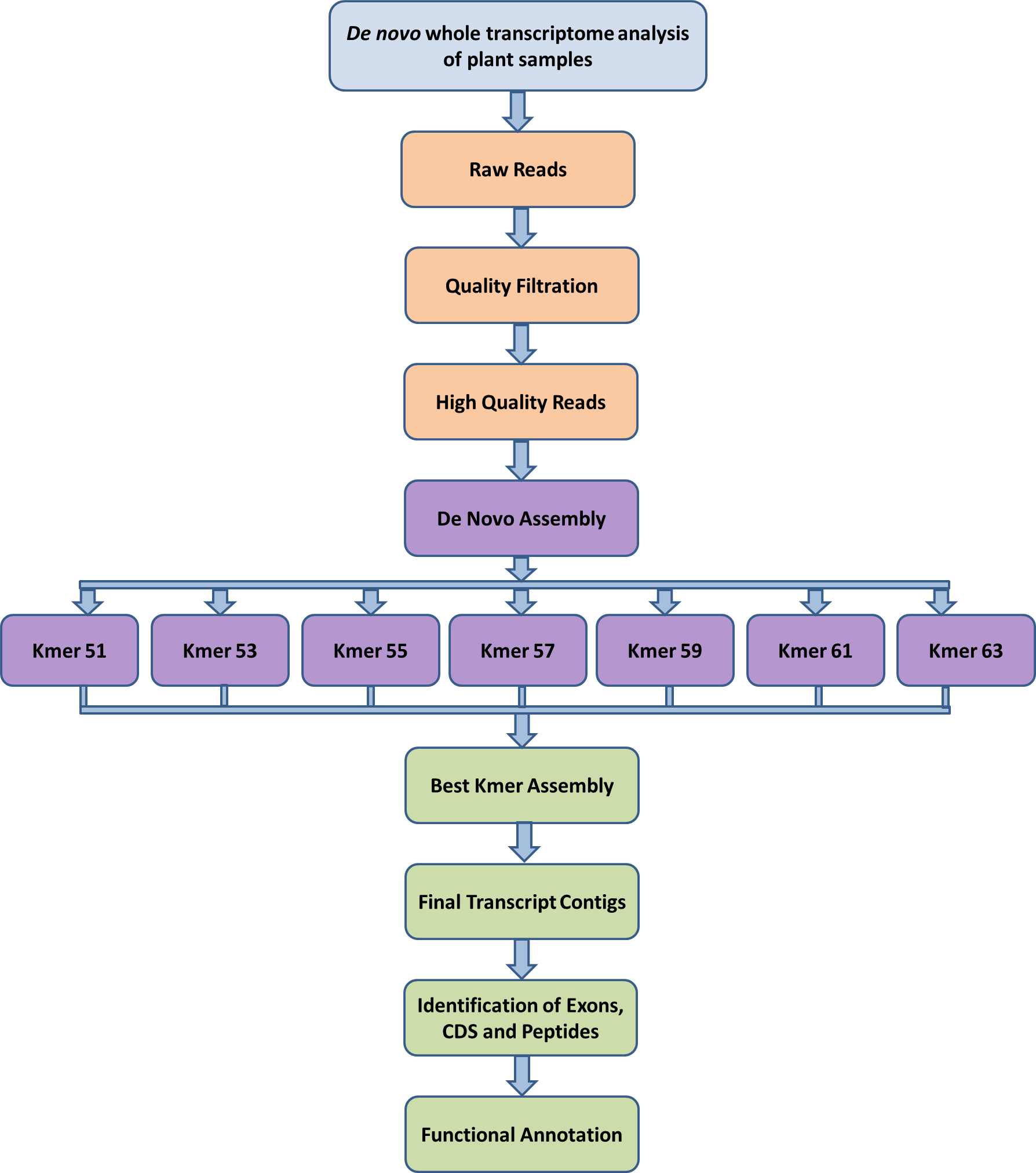

Supplement: S1 Fig — (TIF) [file pone.0144546.s001.tif]
