# Supplementary material for: NGS Transcriptomes and Enzyme Inhibitors Unravel Complexity of Picrosides Biosynthesis in Picrorhiza kurroa Royle ex. Benth
Source: PLoS One. 2015 Dec 11;10(12):e0144546. doi: 10.1371/journal.pone.0144546 (PMC4687646; doi:10.1371/journal.pone.0144546)
Supplement: S1 Table — PKS-25 (Tissue culture grown shoots at 25°C), PKSR (Field grown root), PKSTS (Field grown stolon), PKR-25 (Tissue culture grown roots at 25°C), PKSS (Field grown shoot), PKS-15 (Tissue culture grown shoots at 15°C). (DOCX) [file pone.0144546.s002.docx]

**Supplementary Table 1.** Assembly statistics for *P. kurroa* tissue datasets. PKS-25 (Tissue culture grown shoots at 25°C), PKSR (Field grown root), PKSTS (Field grown stolon), PKR-25 (Tissue culture grown roots at 25°C), PKSS (Field grown shoot), PKS-15 (Tissue culture grown shoots at 15°C)

| **Description** | **PKS-25** | **PKSR** | **PKSTS** | **PKR-25** | **PKSS** | **PKS-15** |
| --- | --- | --- | --- | --- | --- | --- |
| Best Kmer | 51 | 55 | 57 | 51 | 51 | 51 |
| Number of transcript contigs | 39,303 | 20,445 | 28,310 | 26,333 | 21,056 | 37,036 |
| Sum transcript contigs length in bp | 73,560,307 | 27,671,592 | 42,852,936 | 22,897,042 | 30,590,943 | 60,632,658 |
| Max transcript contigs length in bp | 14,624 | 25,087 | 18,983 | 19000 | 30,524 | 16,180 |
| Min transcript contigs length in bp | 524 | 512 | 519 | 163 | 509 | 528 |
| Mean transcript contigs length in bp | 1,871 | 1,353 | 1,514 | 869.51 | 1,453 | 1,637 |
| N50 value in bp | 2,057 | 1,438 | 1,636 | 950 | 1,532 | 1,761 |
| Exons | 50,056 | 22,998 | 33,431 | 39,188 | 25,202 | 46,950 |
| CDS | 39,683 | 20,499 | 28,449 | 26,723 | 21,218 | 37,355 |
