# Supplementary material for: NGS Transcriptomes and Enzyme Inhibitors Unravel Complexity of Picrosides Biosynthesis in Picrorhiza kurroa Royle ex. Benth
Source: PLoS One. 2015 Dec 11;10(12):e0144546. doi: 10.1371/journal.pone.0144546 (PMC4687646; doi:10.1371/journal.pone.0144546)
Supplement: S2 Table — (DOCX) [file pone.0144546.s003.docx]

**Supplementary Table 2.** The paralogs selected for pathway genes in different transcriptomes generated from *P. kurroa* shoot tissues

| **Genes** | **NCBI Accession** | **Highest blast similarity (with NCBI accession) transcript** | | | **Abundance (FPKM)** | | | **Selected transcript ID** | **Highest similarity (with PKSS) of PKS-15 and PKS-25 transcript** | |
| --- | --- | --- | --- | --- | --- | --- | --- | --- | --- | --- |
|  |  | **Transcript ID (Query coverage, Similarity) (Size in bp)** | | |  |  |  |  |  |  |
|  |  | **PKSS** | **PKS-15** | **PKS-25** | **PKSS** | **PKS-15** | **PKS-25** |  | **PKS-15** | **PKS-25** |
| ACTH | *C. roseus*  JF739870.1 (1266 bp) | PKSS_5199 (95, 82) (1215 bp) | PKS-15_14759 (95, 82) (1215 bp) | - | 25.5 | 6.3 | - | PKSS_5199 | 100, 99 | - |
| HMGS | *C. sinensis*  JQ390224.1 (1829 bp) | PKSS_4343 (75, 79)  (1383 bp) | PKS-15_12688 (75, 79) (1383 bp) | PKS-25_16760 (75, 79) (1383 bp) | 133.2 | 20 | 34.2 | PKSS_4343 | 100, 99 | 100, 99 |
| HMGR | *E. pekinensis*  EF062569.1 (2200 bp) | PKSS_2765 (66, 75)  (1695 bp) | PKS-15_3158 (66, 76) (1659 bp) | PKS-25_8607 (67, 76) (1701 bp) | 47.9 | 47.2 | 23 | PKSS_2765 | 87, 75 | 83, 76 |
| MVK | *B. monnieri*  JQ670899.1 (1307 bp) | PKSS_5807 (88, 80) (1164 bp) | PKS-15_15808 (88, 80) (1164 bp) | PKS-25_22471 (88, 80) (1164 bp) | 19.2 | 19.5 | 8.4 | PKSS_5807 | 100, 99 | 100, 99 |
| PMK | *C. roseus*  HM462020.1 (1555 bp) | PKSS_5563 (90, 75) (1419 bp) | PKS-15_27190 (54, 74) (888 bp) | PKS-25_14643 (95, 75) (1521 bp) | 30.3 | 34.4 | 22.4 | PKSS_5563 | 57, 99 | 100, 99 |
| MVDD | *P. ginseng* GQ455989.2 (1254 bp) | PKSS_7386 (99, 780) (1260 bp) | PKS-15_17630 (98, 78) (1239 bp) | PKS-25_19258 (99, 78) (1260 bp) | 33.9 | 18.7 | 19.6 | PKSS_7386 | 98, 99 | 100, 99 |
| IPPI | *I. batatas* DQ150100.1 (1155 bp) | PKSS_14086 (61, 80) (921 bp) | PKS-15_23153 (61, 80) (708 bp) | PKS-25_25624 (61, 81) (708 bp) | 108.2 | 38.8 | 96.9 | PKSS_14086 | 100, 99 | 100, 99 |
| GDS | *V. vinifera* AY351862.1 (1028 bp) | PKSS_17117 (64, 77) (672 bp) | PKS-15_19184 (96, 77) (1241 bp) | PKS-25_19858 (96, 77) (1260 bp) | 26.3 | 12.8 | 12.4 | PKS-15_19184 | 100, 94 | 100, 96 |
| DXPS | *S. rebaudiana* AJ429232.2 (2142 bp) | PKSS_3192 (67, 73) (1461 bp) | PKS-15_3519 (86, 71) (2190 bp) | PKS-25_6679 (86, 71) (2184 bp) | 0 | 25.1 | 19.7 | PKS-15_3519 | 99, 84 | 99, 85 |
| DXPR | *C. acuminata* DQ355159.1 (1823 bp) | PKSS_3705 (74, 75) (1428 bp) | PKS-15_8628 (74, 75) (1428 bp) | PKS-25_12386 (74, 75) (1428 bp) | 34.8 | 23.5 | 126.8 | PKSS_3705 | 100, 99 | 100, 99 |
| ISPD | *G. biloba* DQ102360.1 (1411 bp) | PKSS_16533 (49, 74) (717 bp) | PKS-15_23679 (49, 74) (720 bp) | PKS-25_33913 (49, 74) (720 bp) | 18.2 | 53.9 | 9.3 | PKSS_16533 | 99, 94 | 99, 94 |
| ISPE | *G. biloba* DQ102358.1 (1800) | PKSS_6777 (44, 74) (1221 bp) | PKS-15_9878 (44, 73) (1221 bp) | PKS-25_17580 (44, 73) (1221 bp) | 16 | 32.8 | 39.3 | PKSS_6777 | 100, 99 | 100, 99 |
| MECPS | *G. biloba* AY971576.1 (836 bp) | PKSS_18307 (57, 77) (684 bp) | PKS-15_33598 (57, 77) (684 bp) | PKS-25_33833 (57, 77) (684 bp) | 37.1 | 45.8 | 74.7 | PKSS_18307 | 100, 99 | 100, 99 |
| HDS | *S. rebaudiana* DQ768749.4 (2599 bp) | PKSS_2512 (65, 81) (1701 bp) | PKS-15_3447 (85, 79) (2229 bp) | PKS-25_6066 (85, 79) (2229 bp) | 34.7 | 51.8 | 85 | PKS-15_3447 | 100, 89 | 100, 89 |
| ISPH | *P. kurroa* EF199770.1 (1713 bp) | PKSS_4591 (81, 97) (1389 bp) | PKS-15_12216 (81, 98) (1389 bp) | PKS-25_17704 (81, 93) (1380 bp) | 21.2 | 39.6 | 147.5 | PKSS_4591 | 100, 99 | 100, 92 |
| GS | *C. roseus* JN882024.1 (1770 bp) | - | PKS-15_10718 (72, 74)* (1629 bp) | PKS-25_14891 (71, 73)* (1770 bp) | - | 27.7 | 29.8 | PKS-15_10718 | 100, 83* | |
| G10H | *C. roseus* KF561461.1 (1482 bp) | PKSS_14268 (50, 89) (861 bp) | - | - | 18.9 | - | - | PKSS_14268 | - | - |
| 10HD | *C. roseus*  KF302069.1 (1137 bp) | PKSS_13246 (75, 74) (858 bp) | PKS-15_21110 (97, 74) (1128 bp) | PKS-25_16819 (97, 75) (1128 bp) | 19.3 | 93.3 | 170.4 | PKS-15_21110 | 100, 90 | 100, 99 |
| IS | *C. roseus* JX974564.1 (1167 bp) | PKSS_10371 (93, 66) (1131 bp) | PKS-15_20867 (93, 66) (1197 bp) | PKS-25_20798 (93, 66) (1179 bp) | 68.7 | 14.9 | 82.6 | PKSS_10371 | 99, 99 | 99, 99 |
| CPM | *M. truncatula* DQ335800.1 (1719 bp) | PKSS_3827 (81, 68) (1509 bp) | PKS-15_11131 (81, 68) (1509 bp) | PKS-25_16069 (81, 86) (1509 bp) | 112.2 | 70 | 69 | PKSS_3827 | 100, 99 | 100, 99 |
| UGT | *P. kurroa* JQ996408.1 (1422 bp) | PKSS_3392 (99, 98) (1428 bp) | PKS-15_13757 (100, 98) (1428 bp) | PKS-25_22805 (84, 99) (1428 bp) | 73.4 | 19.7 | 61.9 | PKSS_3392 | 99, 99 | 98, 71 |
| ALD | *A. thaliana* AY056398.1 (1709 bp) | PKSS_4439 (85, 70) (1515 bp) | - | - | 52.4 | - | - | PKSS_4439 | - | - |
| F3D | *G. max* FJ770474.1 (1134 bp) | PKSS_5831 (88, 37) (1011 bp) | PKS-15_22234 (85, 37) (1011 bp) | PKS-25_23477 (85, 38) (1011 bp) | 186.4 | 12 | 11.4 | PKSS_5831 | 100, 89 | 100, 96 |
| 2HFD | *G. max* AB154415.1 (960 bp) | PKSS_8401 (46, 69) (969 bp) | PKS-15_23748 (46, 69) (909 bp) | PKS-25_30081 (46, 69) (909 bp) | 35 | 15.5 | 10.1 | PKSS_8401 | 93, 99 | 93, 99 |
| UPD | *M. truncatula* XM_003600989.1 (1398 bp) | PKSS_6747 (72, 77) (1155 bp) | PKS-15_18082 (72, 77) (1314 bp) | PKS-25_22449 (72, 77) (1209 bp) | 12.7 | 0.2 | 113.9 | PKSS_6747 | 95, 90 | 95, 90 |
| UGD | *G. hirsutum* EU817581.1 (1311 bp) | PKSS_6772 (77, 82) (1197 bp) | PKS-15_15606 (76, 82) (1317 bp) | PKS-25_17015 (64, 76) (1296 bp) | 24.2 | 21.7 | 20.3 | PKSS_6772 | 100, 99 | 100, 99 |
| SQM | *T. cacao* XM_007048297.1 (1874 bp) | PKSS_10700 (67, 84) (1077 bp) | PKS-15_9913 (74, 75) (1590 bp) | PKS-25_11305 (90, 79) (1590 bp) | 41.8 | 30 | 43.9 | PKSS_10700 | 100, 99 | 100, 99 |
| ACT | *E. sagittatum* KJ010528.1 (1564 bp) | - | PKS-15_12041 (99, 35)* (1404 bp) | PKS-25_15966 (99, 35)* (1404 bp) | - | 51.2 | 31.5 | PKS-15_12041 | 100, 100* | |
| DAHPS | *N. tabacum* M64261.1 (1928 bp) | PKSS_3016 (73, 81) (1599 bp) | PKS-15_12601 (73, 81) (1599 bp) | PKS-25_18536 (73, 81) (1506 bp) | 152.8 | 31.6 | 30.4 | PKSS_3016 | 100, 98 | 92, 86 |
| DQS | *V. vinifera* FJ604857.1 (1371 bp) | PKSS_4974 (80, 79) (1359 bp) | PKS-15_15388 (80, 79) (1359 bp) | PKS-25_20294 (80, 79) (1359 bp) | 31.3 | 15.4 | 34.9 | PKSS_4974 | 100, 99 | 100, 98 |
| CAM | *V. planifolia* AY555144.1 (1219 bp) | PKSS_16915 (43, 70) (858 bp) | PKS-15_ 22575 (43, 67) (1113 bp) | PKS-25_ 26225 (43, 70) (1050 bp) | 12.2 | 6.6 | 14.4 | PKSS_16915 | 84, 74 | 100, 97 |
| QSD | *M. truncatula* XM_003608150.1 (1521 bp) | PKSS_8245 (78, 71) (1245 bp) | PKS-15_9901 (83, 71) (1545 bp) | PKS-25_12191 (98, 66) (1545 bp) | 25.1 | 22.1 | 9.9 | PKSS_8245 | 100, 99 | 100, 99 |
| SK | *O. sativa* AB188834.1 (927 bp) | PKSS_11709 (64, 67) (873 bp) | PKS-15_24906 (64, 66) (873 bp) | PKS-25_35453 (64, 67) (663 bp) | 44.3 | 13.1 | 27.8 | PKSS_11709 | 99, 99 | 75, 99 |
| EPSPS | *Z. japonica* GU256772.1 (1268 bp) | PKSS_6421 (88, 74) (1158 bp) | PKS-15_11760 (98, 73) (1536 bp) | PKS-25_14110 (95, 74) (1557 bp) | 54 | 23.3 | 39.7 | PKSS_6421 | 99, 92 | 100, 99 |
| CS | *V. vinifera* FJ604855.1 (1312 bp) | PKSS_4687 (87, 81) (1320 bp) | PKS-15_12629 (87, 81) (1326 bp) | PKS-25_17043 (87, 81) (1326 bp) | 42.1 | 35.8 | 62.5 | PKSS_4687 | 99, 97 | 99, 97 |
| CM | *A. thaliana* L47355.1 (1006 bp) | PKSS_11804 (58, 66) (963 bp) | PKS-15_29160 (53, 66) (933 bp) | PKS-25_26162 (58,66) (963 bp) | 26.1 | 23.6 | 70.8 | PKSS_11804 | 96, 99 | 100, 99 |
| APD | *M. truncatula* XM_003610396.1 (1443 bp) | PKSS_7746 (62, 76) (1020 bp) | PKS-15_17733 (65, 74) (1182 bp) | PKS-25_21892 (64, 76) (1182 bp) | 18.3 | 5.7 | 6.4 | PKSS_7746 | 100, 95 | 100, 99 |
| TAT | *G. max* DQ003328.1 (1709 bp) | - | PKS-15_27179 (43, 74)* (804 bp) | PKS-25_27461 (43, 74)* (804 bp) | - | 13.5 | 15 | PKS-15_27179 | 99, 100* | |
| PAL | *P. kurroa* JQ996410.1 (2312 bp) | PKSS_1122 (91, 82) (2133 bp) | PKS-15_14278 (55, 84) (1296 bp) | PKS-25_7770 (91, 82) (2133 bp) | 78.1 | 18.8 | 11.3 | PKSS_1122 | 60, 99 | 100, 98 |
| C4H | *C. sinensis* AY641731.2 (1352 bp) | PKSS_7335 (86, 79) (1341 bp) | PKS-15_15422 (86, 79) (1518 bp) | PKS-25_10021 (86, 79) (1947 bp) | 193.5 | 246.6 | 167.9 | PKSS_7335 | 99, 99 | 99, 99 |
